# Deep Learning Methods for Automatic Identification of Male and Female Chickens in a Cage-Free Flock

**DOI:** 10.3390/ani15131862

**Published:** 2025-06-24

**Authors:** Bidur Paneru, Ramesh Bahadur Bist, Xiao Yang, Anjan Dhungana, Samin Dahal, Lilong Chai

**Affiliations:** Department of Poultry Science, College of Agricultural & Environmental Sciences, University of Georgia, Athens, GA 30602, USA

**Keywords:** poultry housing, rooster monitoring, animal behavior, precision farming, deep learning

## Abstract

Rooster behavior and activity are critical for egg fertility and hatchability in broiler and layer breeder houses. Desirable roosters are expected to have good leg health, reach sexual maturity, be productive, and show less aggression toward females during mating. However, not all roosters are desirable, and low-productive roosters should be removed and replaced. The objectives of this study were to apply an object detection model based on deep learning to identify hens and roosters based on phenotypic characteristics, such as comb size and body size, in a cage-free (CF) environment, and to compare the performance metrics among the applied models. Six roosters were mixed with 200 Lohmann LSL Lite hens during the pre-peak phase in a CF research facility and were marked with different identifications. Deep learning methods, such as You Only Look Once (YOLO) models, were innovated and trained (based on a comb size of up to 2500 images) for the identification of male and female chickens based on comb size and body features. The performance matrices of the YOLOv5u and YOLOv11 models, including precision, recall, mean average precision (mAP), and F1 score, were statistically compared for hen and rooster detection using a one-way ANOVA test at a significance level of *p* < 0.05. For rooster detection based on comb size, YOLOv5lu, and YOLOv11x variants performed the best among the five variants of each model, with YOLOv5lu achieving a precision of 87.7%, recall of 56.3%, and mAP@0.50 of 60.1%, while YOLOv11x achieved a precision of 86.7%, recall of 65.3%, and mAP@0.50 of 61%. For rooster detection based on body size, YOLOv5xu, and YOLOv11m outperformed other variants, with YOLOv5xu achieving a precision of 88.9%, recall of 77.7%, and mAP@0.50 of 82.3%, while YOLOv11m achieved a precision of 89.0%, recall of 78.8%, and mAP@0.50 of 82.6%. This study provides a reference for automatic rooster monitoring based on comb and body size and offers further opportunities for tracking the activities of roosters in a poultry breeder farm for performance evaluation and genetic selection in the future.

## Simple Summary

Desirable roosters are healthy, mature, productive, active, and gentle toward hens during mating, while undesirable ones should be replaced for better egg fertility in both broiler and layer breeding. This study used deep learning models to automatically detect hens and roosters based on comb and body size in a cage-free laying hen facility. Model performance varied among model variants, with the best model resulting in 88% precision in detecting hens and roosters based on comb size and 89% precision in detecting roosters based on body size measurements. This study provides a baseline for automatic rooster detection and offers an opportunity to further improve detection precision by integrating more features of roosters, such as comb size, color, plumage condition, body weight, and body posture. It also provides an opportunity to monitor the activities of roosters on the breeder farm for genetic selection.

## 1. Introduction

The performance and activities of male breeders (roosters) are critical for egg fertility and hatchability in commercial layer and broiler breeding houses [1,2]. In a study, researchers found positive correlations between rooster body weight and both the laying intensity of fertilized eggs and the hatchability percentage of chicks [2]. A review by Kingor [1] underscores the importance of how the physical conditions and management of roosters can directly impact reproductive outcomes. Desirable roosters are expected to have strong legs, high sexual maturity, productive performance, and less aggression toward female breeders during mating. The selection of roosters with desirable characteristics has been a huge challenge in both the broiler and layer breeder industries. Some challenges arise from roosters maturing sexually earlier than their female counterparts. Research has reported that roosters display more aggressive behaviors during mating, while courtship behaviors are virtually absent before mating [3,4,5,6,7]. The sexual behavior of broiler breeder roosters has been reported to be rough, such as pecking, chasing females, and forcing copulations [6,7,8]. This may be one of the reasons why females have severe wounds on their backs and the back of their heads [6]. In a study that looked at mating behavior, only a maximum of 44% of the matings were successful between the ages of 20 and 28 weeks, and 80% were forced matings [9]. These earlier findings show that there is a need to find a way to automatically detect roosters from hens, track their behavior and activities, and remove those with undesirable behaviors.

Rooster activities, such as locomotion and daily trajectory, are critical for egg fertility and hatchability in broiler and layer breeding houses. However, roosters do not always perform as expected. The breeding program may use the secondary sex characteristics of roosters, such as comb, plumage, and wattle development, for selecting roosters. Researcher Wei and the team [10] used an intelligent video surveillance system with color-calibrated cameras and the YOU ONLY LOOK ONCE (YOLO) YOLOv4 algorithm to monitor broiler chickens’ comb color, which changes as they grow and can indicate health issues. The system provides real-time, accurate health monitoring, outperforming traditional manual methods and earlier artificial intelligence models. The YOLOv4 model used in this study for comb detection achieved a precision of 90% and a recall of 89%. The model was highly accurate in identifying chicken combs and had a strong ability to detect combs correctly under various lighting conditions and zoom levels. However, it faced challenges in distinguishing small combs or those positioned too far away from the images. This shows that the comb is an important physical characteristic of chickens, especially roosters, to differentiate them from hens, and could be used as a proxy for detecting hens and roosters.

Rooster behaviors, such as mating and movement, can indicate their productivity and welfare. However, manually identifying these behaviors from recorded videos is challenging and time-consuming, often leading to issues such as cognitive bias and fatigue. Deep learning techniques offer a potential solution to these challenges, and properly trained models can effectively detect roosters in complex environments, such as CF poultry houses. To address object detection problems, Joseph Redmon first introduced the YOLO (You Only Look Once) model in 2015 [11], emphasizing real-time processing, and it remains one of the leading object detection models today. The YOLO family of models is a widely used real-time object detection framework that is known for its speed and accuracy. Unlike traditional object detection methods, which employ region proposal networks or sliding window approaches, YOLO treats object detection as a single regression problem, directly predicting bounding boxes and class probabilities from an image in a single pass through a neural network [11]. These models have been effectively used in security and surveillance, sports, retail, automotive, healthcare, and agriculture. In addition to this area, YOLO models have been rapidly applied in the poultry industry and have become part of precision poultry farming.

Precision poultry farming is a hot topic in the poultry industry, and the application of deep learning techniques has gained significant attention. Object detection models alone or in combination with other deep learning models have been used for various purposes, such as detecting brown and dead hens in a CF litter floor [12,13] and detecting applied behaviors, such as dustbathing and perching [14,15], in CF housing. A study demonstrated that the YOLOv3 model can accurately detect and recognize six behaviors of egg breeders with a mean average precision (mAP) of 92.09%, achieving high recognition rates for mating (94.72%), standing (94.57%), and feeding (93.10%) among others [16]. In addition to YOLOv3, researchers have also utilized the YOLOv4 algorithm to automatically detect feeding and aggressive behaviors in broiler chickens, where the model achieved high mAP values of 99.98% for feeding and 99.40% for aggressive behaviors [17]. Researchers also developed an algorithm using a Convolutional Neural Network (CNN) and image processing to estimate the feeding time of individual broilers and achieved an overall accuracy of 87.30% in estimating feeding time per visit to the feeding pen [18]. Another application of the YOLOv4 model was found in measuring comfort behavior, such as dustbathing in an experimental aviary, and it was found that the models struggled to accurately classify dustbathing hens, achieving only 28.2% and 31.6% precision for YOLOv4-tiny and YOLOv4, respectively [19]. In addition to behavior detection, research has been conducted combining an autoencoder and YOLOv6 for classification and disease detection [20], and a YOLO-based model has been used for the automatic detection of broiler pathological phenomena [21], along with the detection and tracking of broiler flock movement in a chicken coop [22]. The YOLOv5s (small) model was used to monitor individual chickens for early disease detection and achieved 96% precision in detecting and characterizing chickens by age [23].

Currently, there is no automated system to identify hens and roosters in a CF facility. The objectives of this study were to apply an object detection model based on deep learning to identify hens and roosters based on phenotypic characteristics, such as comb size and body size, in a cage-free (CF) environment, and to compare the performance metrics among the applied models.

## 2. Materials and Methods

### 2.1. Experimental Setup

**Figure 1:**
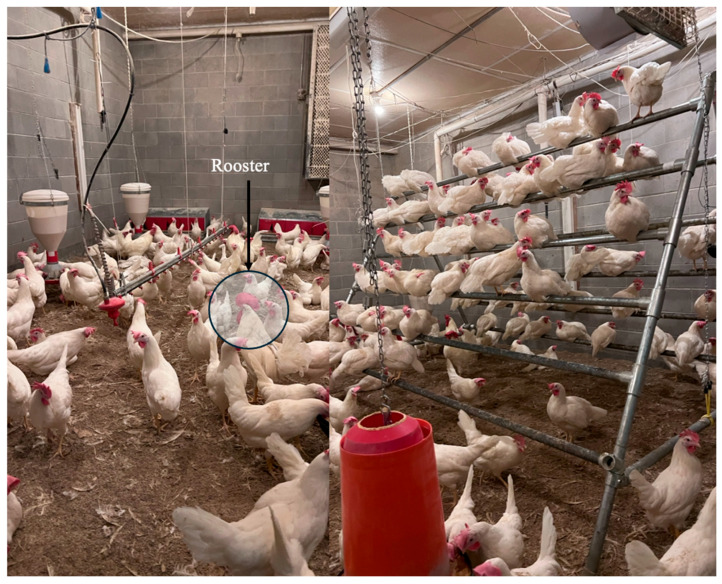
Cage-Free (CF) Research facility housing six marked roosters with 200 laying hens during the pre-peak phase (week 19–27).

About 800 chicks (Lohmann LSL Lite) were raised from week 1 to week 27 for laying hen research in four research CF facilities at the University of Georgia, Athens, GA, USA. Six birds were found to be male (rooster) out of 800 chicks, which were marked with a black color marker (Livestock Marker Crayon) as Male 1 to 6 on the back when the birds reached 18–19 weeks at the University of Georgia, USA. Although the recommended ratio of males to females in laying hen breeding is 1:10, we had an obligation to use 0.3:10 (6 roosters: 200 hens) as we could not bring new roosters to the research facility according to the UGA research policy. Although the ratio is lower than recommended, we did not expect it to impact our results, as the study only focused on detecting roosters and hens from the flock rather than pure breeding. The research facility was designed with provisions for litter floors, perches, and nest boxes to allow hens to perform natural behaviors such as perching, dustbathing, and foraging. Each study room measured 7.3 m in length, 6.1 m in width, and 3 m in height (Figure 1). Bird management (free access to feeders, drinkers, perches, nest boxes for 24 h a day, and lighting) in the facility was maintained according to the Lohmann LSL Lite management guidelines [24]. From the first day of the study, the floor was lined with approximately 5 cm of pine shavings as a bedding material for the birds. Environmental conditions, including indoor temperature, relative humidity, light duration, light intensity, and ventilation rates, were automatically monitored and managed using the Chore-Tronic Model 8 controller (Chore-Time Equipment, Milford, IN, USA). Birds were provided 13, 14, and 15 h of light during weeks 19, 20, and 21, respectively, during (pre-peak), and 16 h of light from week 22 to week 27 (layer phase 1). Light intensity was maintained at 10–15 lx, and room temperature was maintained at 18–20 °C from week 19 to week 27, as per the Lohmann LSL Lite management guidelines [24]. The feed (soy-corn) was formulated following the Lohmann LSL-LITE management nutrition guidelines [24]. The feed was mixed monthly at the UGA feed mill to ensure freshness, preserve quality, and prevent mold growth. The animal care and management procedures used in this study were approved by the Institutional Animal Care and Use Committee (IACUC) of the University of Georgia.

### 2.2. Poultry Image Data Collection

To monitor the marked roosters in the research facility, six night-vision network cameras (PRO-1080MSB, Swann Communications USA Inc., Santa Fe Springs, LA, USA) were mounted on the ceiling ∼3 m above the litter floor to record videos 24 h a day. The cameras were installed at a top-down 90° field of view with a visual field of coverage area of 6 m × 6 m. We chose night-vision network cameras over regular cameras to detect birds’ activities and behavior during the nighttime. However, to make the data pre-processing appropriate and reduce unnecessary video files, the videos of the birds’ activities were stored and backed up daily. Dust emission was one of the major challenges during the study, which covered the surface of the camera hanging on the ceiling and resulted in blurred videos. To avoid dust, the camera lenses were cleaned every alternate day while taking care of the hens in the morning hours. The recorded videos were stored in. avi format at a resolution of 1920 × 1080 pixels and a frame rate of 15 Frames Per Second (FPS) using a DVR-4580 digital video recorder (Swann Communications USA Inc., Santa Fe Springs, LA, USA). To ensure the integrity and security of the data during the process of data storage and backup, it was always done in a university network, creating offline storage backups and periodic restores from backups to ensure that they work.

### 2.3. Image Labeling and Data Pre-Processing

**Figure 2:**
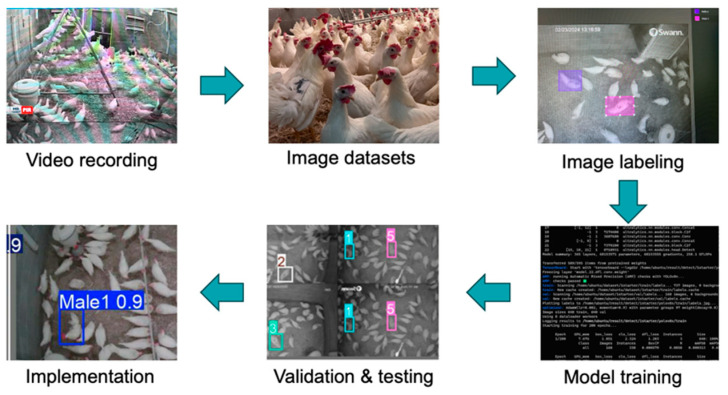
The workflow for hen and rooster detection using the YOLOv5u and YOLO11 models (i.e., data collection, image labeling, training, validation, testing, and implementation).

Video datasets obtained from research facilities were converted into individual .jpg image files at a frame rate of 15 FPS using the Free Video to JPG Converter App (ver. 5.0). The resulting image datasets were manually filtered and stored based on the presence of at least one rooster in each image. The images were randomly selected for training, validation, and testing. A total of 100 chicken comb images and 2500 chicken body images were used to train the deep-learning models to quantify the differences between hens and roosters, where 70% of the images were used for training, 20% for validation, and 10% for testing. For hen and rooster detection based on comb size and rooster detection based on body size, image labeling was performed using the free website makesense.ai. Each image with hens and roosters was labeled with a bounding box of the corresponding hen’s comb as a hen and rooster’s comb as a rooster for hen and rooster detection based on comb size, while each image with roosters was labeled with a bounding box corresponding to each rooster number as Male1, Male2, Male3, Male4, Male5, and Male6 for roosters that were marked 1, 2, 3, 4, 5, and 6 to detect roosters based on body size. The workflow for hen and rooster detection based on comb size and rooster detection based on body size using both the YOLOv5u and YOLOv11 models is shown in (Figure 2).

### 2.4. Model Architecture: YOLOv5u vs. YOLOv11

The YOLOv5u model is an advanced version of the standard YOLOv5 model developed by Ultralytics [25]. It has five model variants: nano (nu), small (su), medium (mu), large (lu), and extra-large (xu). YOLOv5u integrates an anchor-free, abjectness-free split head, a feature previously introduced in the YOLOv8 models, which significantly improves the accuracy-speed tradeoff in object detection tasks. Given the practical results and their derived features, YOLOv5u offers an efficient alternative for robust solutions in both research and practical applications. We used this model to compare its performance with the latest version of the object detection model, YOLO11. YOLOv5u confirmed a more flexible and adaptive detection mechanism, consequently enhancing its performance in diverse scenarios. The major advantage of using YOLOv5u is that it balances accuracy and speed, which often pull in opposite directions. YOLOv5u offers a calibrated balance, ensuring real-time detection without compromising accuracy. YOLOv5u also provides a variety of pre-trained models, which ensures a model fine-tuned for the unique challenge, but not just for a particular scenario.

**Figure 3:**
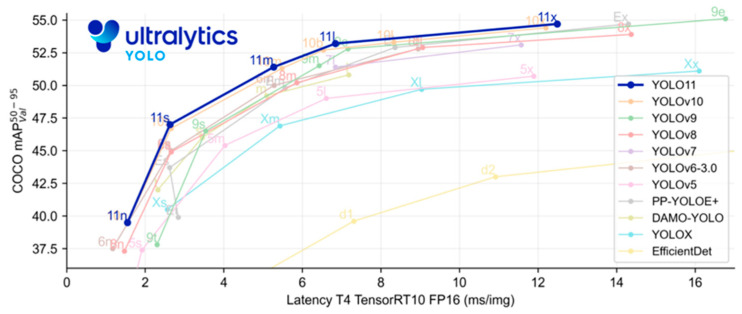
Comparison of YOLO11 model performance (mAP 50–95) on the COCO dataset against earlier versions of YOLO models, as explained by Ultralytics [26].

YOLOv11 represents a cutting-edge advancement in object detection, offering a balance of speed, accuracy, and efficiency by incorporating innovations such as C3k2 blocks for feature extraction and C2PSA attention mechanisms to emphasize critical areas within images [26]. It has five model variants: nano (n), small (s), medium (m), large (l), and extra-large (x). YOLO11 effectively balances the speed-accuracy tradeoff. The model comprises three major parts: the backbone, neck, and head. The backbone acts as the primary feature extractor, which uses a convolutional neural network (CNN) to transform raw image data into multi-scale feature maps. The neck part acts as an intermediate processing stage, integrating specialized layers to aggregate and enhance feature representations across different scales. The head functions as a prediction mechanism, gathering the final outputs for object localization and classification based on the refined feature maps. Comparison of YOLO11 model performance (mAP @0.50–0.95) on the COCO dataset against earlier versions of YOLO models, as explained by Ultralytics, [26] is presented in (Figure 3) below.

### 2.5. Model Evaluation Metrics

To evaluate the model performance, we calculated the precision, recall, mean average precision (mAP), and F1 score as the key indicators. Precision refers to how well the model correctly identifies hens and roosters without including incorrect predictions. It shows the model’s predictions that are correct. For example, if the model predicts 100 hens in a video frame, but only 90 of them are hens and the remaining ten are rooster or background objects incorrectly identified as hens, then the precision is 90%.

(1) $$Precision=\frac{True\;Positive}{True\;Positive+False\;positive}$$

Recall indicates the model’s capability to correctly predict all relevant positive instances within the datasets. It is the ratio of true positive predictions to the total number of positive predictions, such as detected hens and roosters, to the total number of actual positive instances in the dataset.

(2) $$Recall=\frac{True\;Positive}{True\;Positive+False\;Negative}$$

Mean average precision (mAP) is a key metric used to evaluate how well the model detects both hens and roosters across different scenarios. It reflects the model’s overall accuracy by combining both precision and recall across multiple detection thresholds, such as Intersection Over Union (IoU), which measures how much the predicted bounding boxes overlap with the ground truth boxes. For example, when using an IoU threshold of 0.50, a predicted box is considered correct if it overlaps at least 50% with the actual location of the hens and rooster.

(3) $$mAP=\frac{\sum_{i-1}^{C}APi}{C}$$

In this equation, *AP_i_* signifies the average precision of the *ith* category, and *C* represents the total number of categories.

The F1 score is an important measure for evaluating the accuracy of the model in detecting hens and roosters. It represents the balance between precision (how many identified birds are correctly labeled) and recall (how many actual birds the model successfully detects), which is calculated using their harmonic mean. The F1 score was calculated using Equation 4. A higher F1 score indicates that the model is not only good at correctly identifying hens and roosters but also at detecting them in the scene. For example, an F1 score of 100% means that all hens and roosters were detected perfectly, with no missed detections.

(4) $$F1\;Score=\frac{2\times Recall\times Precision}{Recall+Precision}$$

## 3. Results and Discussions

### 3.1. Performance Metrics Comparison: Hen and Rooster Detection (Comb Size)

The models’ performance metrics, such as precision, recall, mAP, and F1 score across YOLOv5u and YOLOv11 models, were compared using one-way ANOVA at a 5% significance level for hen and rooster detection based on the comb size. There was no convincing evidence to suggest a difference in the performance metrics between the YOLOv5u and YOLOv11 models. However, YOLOv5lu and YOLOv11x variants performed the best among the five variants of each model. Their performance was similar, with YOLOv5lu achieving a precision of 87.7%, recall of 56.3%, mAP@0.50 of 60.1%, and F1-score of 65%, while YOLOv11x achieved a precision of 86.7%, recall of 65.3%, mAP@0.50 of 61%, and F1-score of 58%. The inclusive evaluation of various YOLOv5u and YOLOv11 models with their respective model variants, nano (n), small (s), medium (m), large (l), and extra-large (x), in terms of their efficiency in detecting hens and roosters in a CF facility, is given below (Table 1).

**Figure 4:**
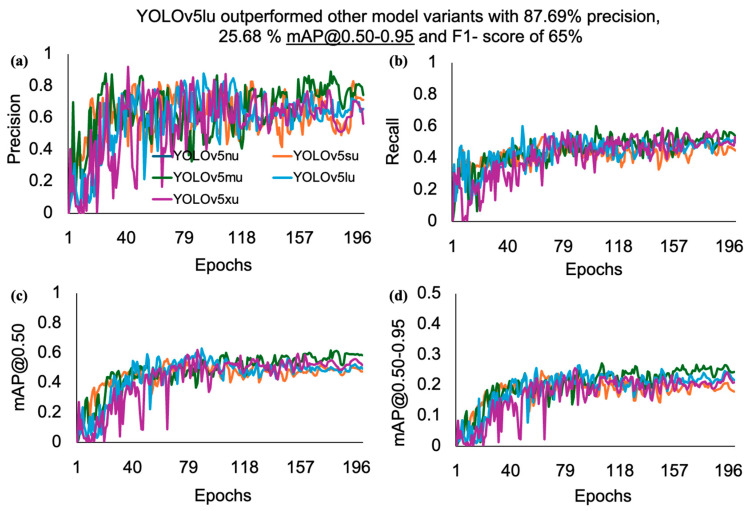
Performance metrics such as (**a**) precision, (**b**) recall, (**c**) mAP@0.50, and (**d**) mAP@0.50-0.95, of the YOLOv5u model versus the number of epochs for detecting hens and roosters based on comb size in a CF facility. Where mAP refers to mean average precision.

The detailed analysis offers thoughtful insights into each model’s performance, easing comparisons across various dimensions. After analyzing the results, it was clear that the YOLOv5lu model outperformed all other examined variants of YOLOv5u, with minimal variations across the performance metrics. A closer examination of its outcomes showed an impressive precision of 87.7%, recall of 56.3%, mAP@0.50 of 60.1%, and F1 score of 65% as the number of epochs increased (Figure 4). The performance metrics of YOLOv5u increased as the model size increased from small (s) to medium (m) and reached the highest performance at the large (l) variant, while the performance metrics slightly decreased as the model size increased from large (l) to extra-large (x). The performance results of YOLOv5lu in detecting hens and roosters were lower than the performance of comb detection in chickens by [10], achieving a precision of 90% and a recall of 89% with the YOLOv4 algorithm. This could be the reason for the relatively small sample size (100 vs. 860 images) and multiple classes (hen and rooster vs. comb) compared with the [10]. The precision of YOLOv5lu (87.7%) in our study was slightly higher than that of the result of [18] in estimating feeding time per visit of broilers to the feeding pen, which achieved an overall accuracy of 87.30%. Our study resulted in higher precision than a previous study that integrated deep learning and fuzzy inference systems to evaluate broiler activities, achieving accuracies of 85.2% for identifying broiler types, 79.2% for identifying body parts, and 50.8% for recognizing behaviors [27].

**Figure 5:**
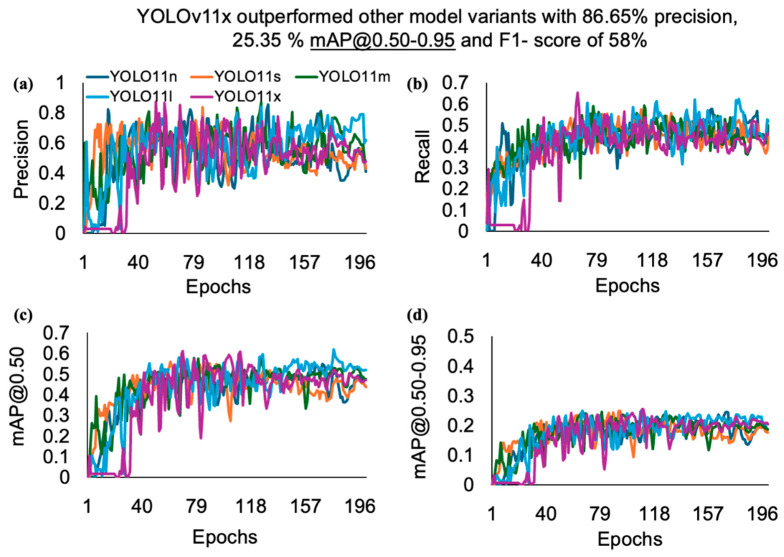
Performance metrics such as (**a**) precision, (**b**) recall, (**c**) mAP@0.50, and (**d**) mAP@0.50-0.95, of the YOLOv11 model versus the number of epochs for detecting hens and roosters based on comb size in a CF facility. Where mAP refers to mean average precision.

When comparing the performances of the YOLOv11 variants, the YOLOv11x model outperformed all other variants, with small variations across the performance metrics. A closer look at its outcomes underscores a precision of 86.7%, a recall of 65.3%, and mAP@0.50 of 61% as the number of epochs increased (Figure 5). The performance metrics of the YOLOv11 variants varied among performance metrics and model variants, such as the precision, which decreased as the model size increased from nano (n) to small (s), increased as the model size increased from small (s) to medium (m), and decreased again from medium (m) to large (l), and finally reached the highest performance in extra-large (x). The YOLOv5lu and YOLO11x variants’ mAP@0.50 score of (60.08% and 61.94%) for hen and rooster detection further highlight the ability of these models to detect hens and rooster instances with a high confidence score compared with other variants of the YOLOv5u and YOLO11 models used. YOLOv5nu exhibited the lowest performance metrics compared to the other variants of the YOLOv5u and YOLOv11 models. It achieved a precision of 83.1%, a recall of 53.3%, mAP@0.50 of 55.9%, and F1 score of 53%. Increasing the sample size, including images throughout the life cycle, and integrating other features of hens and roosters, such as body size, could further increase the detection precision and recall, as our image datasets were only gathered during the pre-peak phase, which could have affected the results.

### 3.2. Performance Metrics Comparison: Rooster Detection (Body Size)

The models’ performance metrics, such as precision, recall, mAP, and F1 score across YOLOv5u and YOLOv11 models, were compared using one-way ANOVA at a significance level of *p* < 0.05 for detection. There was no convincing evidence of a significant difference in the performance metrics between the YOLOv5u and YOLOv11 models. However, the YOLOv5xu and YOLOv11m variants performed the best among the five variants of each model. Their performance was similar, with YOLOv5xu achieving a precision of 88.9%, recall of 77.7%, mAP@0.50 of 82.3%, mAP@0.50–0.95 of 56.1%, and F1 score of 78%, whereas YOLOv11m had a precision of 89%, recall of 78.8%, mAP@0.50 of 82.6%, mAP@0.50–0.95 of 55.6%, and F1 score of 78%. A comprehensive evaluation of various YOLOv5u and YOLOv11 models with their respective model variants n, s, m, l, and x in terms of their efficiency in detecting roosters in a CF facility is given below (Table 2).

**Figure 6:**
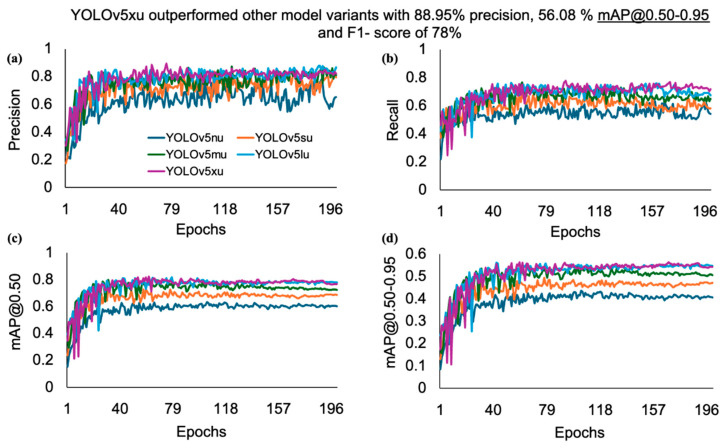
Performance metrics such as (**a**) precision, (**b**) recall, (**c**) mAP@0.50, and (**d**) mAP@0.50-0.95, of the YOLOv5u model versus the number of epochs for detecting roosters based on body size in a CF facility. Where mAP refers to mean average precision.

The detailed analysis offers profound insights into the performance of each model, facilitating comparisons across various dimensions. After analyzing the results, it was clear that the YOLOv5xu model outperformed all other examined variants of YOLOv5u, with minimum variations across the performance metrics. A closer inspection of its outcomes showed a notable precision of 88.9%, recall of 77.7%, mAP@0.50 of 82.3%, mAP@0.50-0.95 of 55.6%, and F1-score of 78% as the number of epochs increased (Figure 6). The performance metrics of the YOLOv5u variants increased as the model size increased from nano (n) to large (l) and reached the highest performance at the extra-large (x) variant.

**Figure 7:**
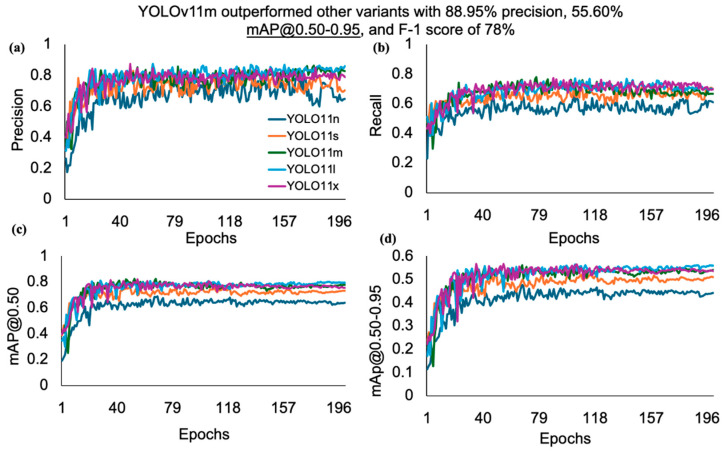
Performance metrics such as (**a**) precision, (**b**) recall, (**c**) mAP@0.50, and (**d**) mAP@0.50-0.95, of the YOLOv11 model versus the number of epochs for detecting roosters based on body size in a CF facility. Where mAP refers to mean average precision.

When comparing the performances of the YOLOv11 variants, the YOLOv11m model outperformed all other variants, with small variations across the performance metrics. A closer examination of its outcomes highlighted a precision of 89%, recall of 78.8%, mAP@0.50 of 82.6%, and F1-score of 78% as the number of epochs increased (Figure 7). The performance metrics of the YOLOv11 variants increased as the model size increased from nano (n) to small (s) and reached the highest performance at the medium (m) variant, after which it started to decline from large (l) to extra-large (x) variants. The YOLOv5xu and YOLOv11m variants’ mAP@0.50 score of (82.3% and 82.6%) for rooster detection based on body size further highlights the ability of these models to detect rooster instances with a high confidence score compared with the other variants of the YOLOv5u and YOLOv11 models used. YOLOv5nu exhibited the lowest performance metrics compared to the other variants of the YOLOv5u and YOLOv11 models. It achieved a precision of 79.1%, recall of 61.7%, mAP@0.50 of 62.8%, mAP@0.50–0.95 of 43.2%, and F1 score of 61%. As mentioned earlier, increasing the image datasets (training datasets: 1750 images) and other features, such as body size, significantly increased the detection performance of both the YOLOv5xu and YOLOv11m models in detecting roosters when compared with the performance of YOLOv5lu and YOLOv11x in detecting hens and roosters based on comb size within this study.

When comparing the F1-confidence curve for hen and rooster detection based on comb size, two variants, YOLOv5lu and YOLOv11x, obtained the highest F1-score of 65% and 62%, respectively, in a CF facility. The F1 score highlights the aptitude of the models at a fine balance between precision and recall across different hen and rooster detections. The highest F1-score obtained by the YOLOv5lu and YOLOv11x variants during hen and rooster detection in a CF facility was relatively lower than the F1-score of detecting roosters based on body size in our study by two model variants, YOLOv5xu, and YOLOv11m. The lower F1-score of the YOLOv5lu and YOLOv11x variants in detecting hens and roosters could be due to the smaller number of images utilized (100 images) than in rooster detection based on body size (2500 images) and the presence of more classes, such as hens and roosters, versus roosters only. The F1 scores across the YOLOv5u models increased as the model size increased from nano (n) to medium (m), reached a maximum in the large (l) variant, and decreased in the extra-large (x) variant. The F1-score of the YOLO11 model also followed the same pattern as that of the YOLOv5u model, where the F1 scores across the YOLOv11 models increased as the model size increased from nano (n) to medium (m), reached a maximum in large (l), and decreased in extra-large (x) variants.

When comparing the F1-confidence curve for rooster detection based on body size, two variants, YOLOv5u and YOLOv11m, obtained the highest F-1 score of 0.78 while detecting roosters in a CF facility. The F1 score highlights the aptitude of the models at a fine balance between precision and recall across different rooster detections. The highest F1-score obtained by the two variants during rooster detection in a CF facility was relatively lower than the F1-score for detecting dustbathing and perching behavior using YOLOv8 models in the CF facility [14,15]. The lower F1-score in rooster detection may be due to the smaller sample size in images of roosters, where the maximum number of possible roosters in one image was only six, while for dustbathing and perching, the number of hens performing such behavior could have gone from 10–50 in a single image, especially in perching detection. Earlier studies have recognized that the higher the F1 score, the better the model performance [28,29,30]. The F1 scores across the YOLOv5u models increased as the model size increased from n to l and reached a maximum in the x variant, while the F1 score of the YOLOv11 model increased from n to s and reached a maximum at m and l variants, then started to decrease in the x variant.

### 3.3. Training and Validation Prediction Result

**Figure 8:**
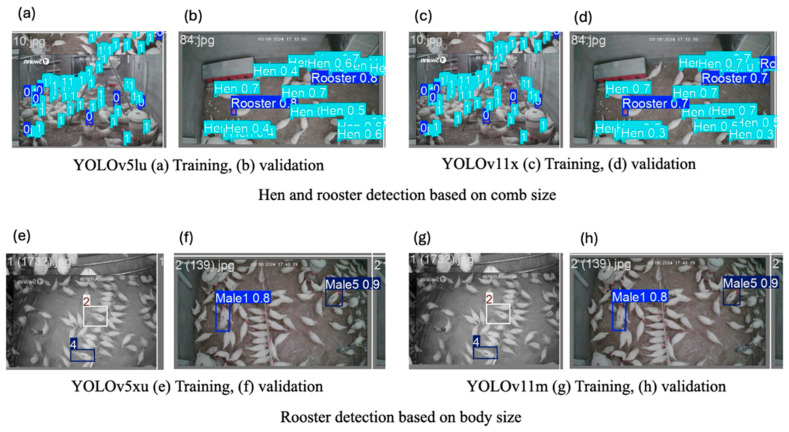
Training and validation of prediction results of YOLOv5lu and YOLOv11x during the detection of hens and roosters. Where, (**a**) YOLOv5lu training, (**b**) YOLOv5lu validation, (**c**) YOLOv11x training, (**d**) YOLOv11x validation based on comb size, and (**e**) YOLOv5xu training, (**f**) YOLOv5xu validation, (**g**) YOLOv11m training, and (**h**) YOLOv11m validation based on body size.

The training and validation prediction results of YOLOv5lu and YOLOv11x during hen and rooster detection based on comb size and YOLOv5xu and YOLOv11m during rooster detection based on body size are presented in Figure 8.

### 3.4. Confusion Matrix Analysis: Hen and Rooster Detection

**Figure 9:**
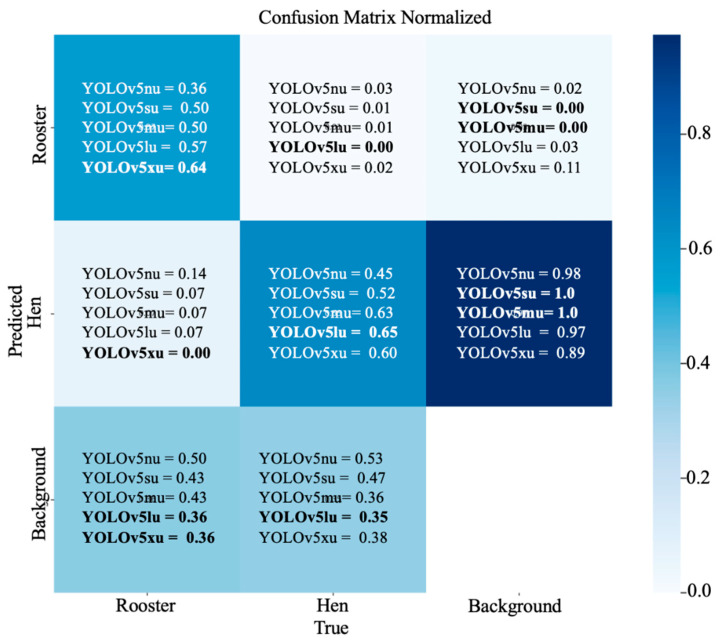
A confusion matrix normalized by the YOLOv5u model during hen and rooster detection based on comb size in a CF environment.

The confusion matrix in the normalized form with the percentage of correct predictions obtained from YOLOv5u during hen and rooster detection based on comb size is shown in (Figure 9). From the confusion metrics, the best-performing model for rooster detection was YOLOv5xu, with 64% of roosters correctly predicted as roosters, followed by YOLOv5lu with 57% of roosters correctly predicted as roosters. The two variants, YOLOv5su and YOLOv5mu, had comparable results, with 50% of roosters correctly predicted as roosters, while YOLOv5nu had the lowest performance, with 36% of roosters correctly predicted as roosters. From the confusion metrics, the best-performing model for hen detection was YOLOv5lu, with 65% of hens correctly predicted as hens, followed by YOLOv5mu, with 63% of hens correctly predicted as hens, and YOLOv5xu, with 60% of hens correctly predicted as hens. YOLOv5su had a correct prediction rate of 52% for hens as hens, while YOLOv5nu had the lowest prediction rate, with only 45% of hens correctly predicted as hens.

**Figure 10:**
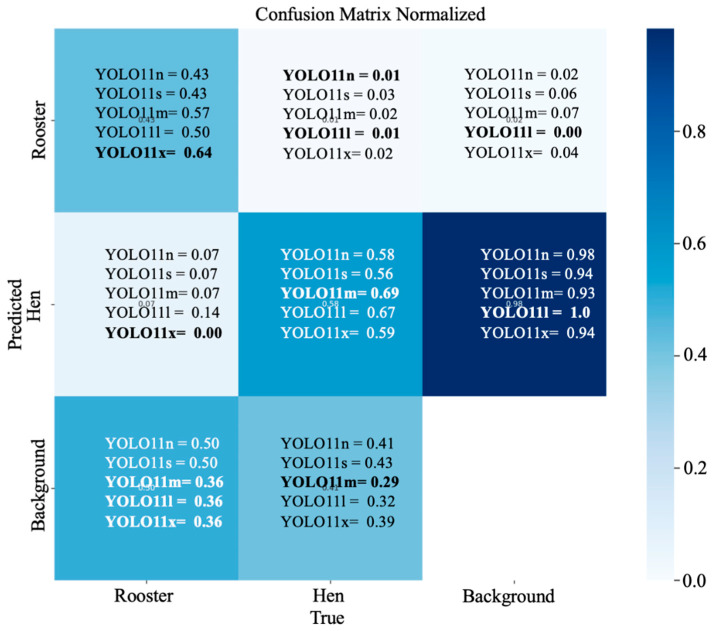
A confusion matrix normalized obtained from the YOLOv11 model during hen and rooster detection based on the comb size in a CF environment.

The confusion matrix in the normalized form with the percentage of correct predictions obtained from the YOLOv11 model during hen and rooster detection based on the comb size is shown in (Figure 10). From the confusion metrics, the best-performing model for rooster detection was YOLOv11x, with 64% of the roosters correctly predicted as roosters, followed by YOLOv11m, with 57% correctly predicted roosters as roosters. YOLOv11l had a 50% correct prediction of roosters as roosters, while YOLOv11n and YOLOv11s had the lowest positive prediction rate, with only 43% of roosters correctly predicted as roosters. From the confusion metrics, the best-performing model for hen detection was YOLOv11m, with 69% of hens correctly predicted as hens, followed by YOLOv11l, with 67% of hens correctly predicted as hens. YOLOv11x and YOLOv11n had a similar performance, with YOLOv11x with a 1% more positive prediction rate, with 59% correctly predicted hens as hens. The lowest-performing model for detecting hens was YOLOv11s, with only 56% of the hens correctly predicted as hens.

### 3.5. Confusion Matrix Analysis: Rooster Detection

**Figure 11:**
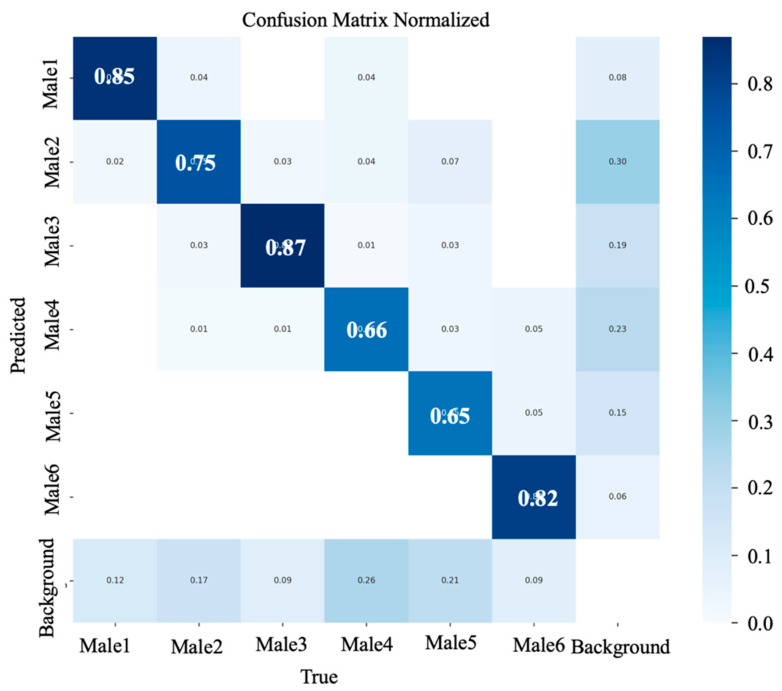
A confusion matrix normalization obtained from the YOLOv5xu model during rooster detection based on body size in a CF environment.

**Figure 12:**
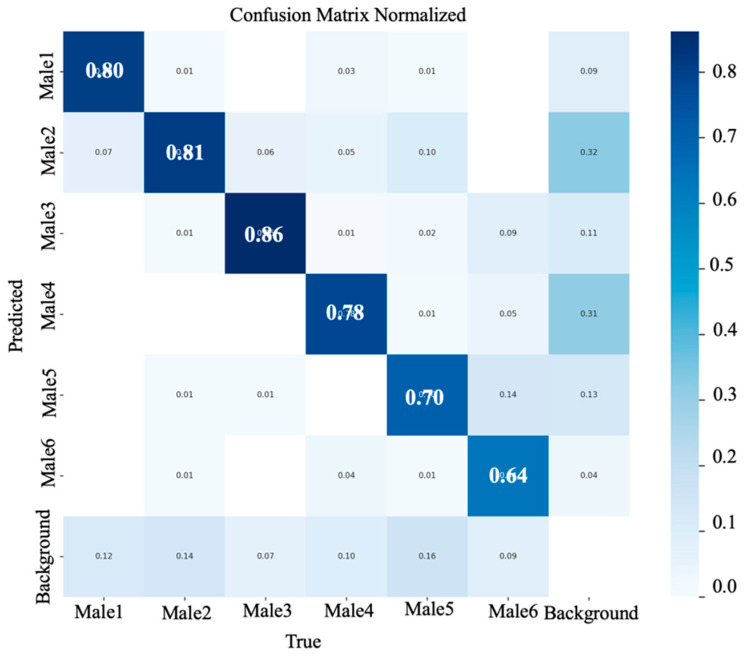
Confusion matrix normalized obtained from the YOLOv11m model during rooster detection based on body size in a CF environment.

The confusion matrix in the normalized form with the percentage of correct predictions obtained from the YOLOv5xu and YOLOv11m during rooster detection based on body size is shown in (Figure 11 and Figure 12). The YOLOv5xu variant showed the highest true positive rate of 0.87 for detecting Male 3, followed by 0.85 for Male 1, 0.82 for Male 6, 0.75 for Male 2, 0.66 for Male 4, and 0.65 for Male 5. The YOLOv11m variants showed the highest True Positive rate of 0.86 for Male 3, followed by 0.81 for Male 2, 0.80 for Male 1, 0.78 for Male 4, 0.70 for Male 5, and 0.64 for Male 6. These results underscore the proficiency of the YOLOv5xu and YOLOv11m models in accurately detecting roosters in a CF facility. A greater number of true positives indicates better model performance in accurately detecting and recognizing positive instances in the dataset [29]. This ability can be further improved by integrating rooster data from early life and a larger sample size, which has huge implications for efficient rooster monitoring and highlights the potential of the YOLOv5xu and YOLOv11m models in precise rooster detection.

## 4. Applications and Benefits of Automatic Hen and Rooster Detection

Automatic rooster detection and tracking activities of roosters have huge benefits for broiler and layer breeding farms, as they provide information that helps to decide whether to keep the rooster in the flock or replace it. One of the benefits of detecting low-performing roosters in the flock is that it can save labor and feed costs associated with managing roosters, and these low-performing roosters can be replaced in time to improve breeding with healthy and productive roosters. Another benefit is the increased fertilization rate to boost hatching profits, as technology helps to monitor the activities of roosters automatically, which does not depend on human observation. The application of this technology also helps in sustainable development, as it helps reduce waste and breeding management.

## 5. Conclusions

This study focused on detecting hens and roosters based on comb size and rooster detection based on body size, using two machine learning models: YOLOv5u and YOLOv11. The research concluded that deep learning-based object detection models, especially YOLOv5lu and YOLOv11x, were effective in detecting hens and roosters based on comb size, while YOLOv5xu and YOLOv11m were effective in detecting roosters based on body size in a CF environment. Although the YOLOv11 models showed robust performance, earlier YOLOv5u models also performed competitively, indicating that newer versions are not always superior. Additionally, utilizing smaller models can reduce the training time and computational demands without compromising performance. This highlights the importance of selecting models based on specific needs and their performance requirements. These findings suggest that similar deep learning models could be applied in broiler and layer breeding programs to detect hens and roosters based on comb size as a first step towards precision rooster selection. Combining other features of roosters, such as body size, plumage condition, and body posture, could further increase the detection performance. The findings set a baseline for automated rooster detection and offer opportunities for performance evaluation and genetic selection in broiler and layer breeder farms.

## Figures and Tables

**Table 1:** Comprehensive evaluation of YOLOv5u and YOLOv11 models for hen and rooster detections. Note: Model variants with bold numbers under evaluation metrics represent the best model within each model.

Models	Precision (%)	Recall (%)	mAP@0.50 (%)	mAP@0.50–0.95 (%)	F1-Score (%)
YOLOv5nu	83.08	53.25	55.93	24.72	53.00
YOLOv5su	83.08	53.17	55.93	24.72	61.00
YOLOv5mu	87.24	64.55	61.33	25.54	65.00
**YOLOv5lu**	**87.69**	**56.33**	**60.08**	**25.68**	**65.00**
YOLOv5xu	87.38	68.58	58.73	24.25	58.00
YOLOv11n	85.33	57.70	54.83	24.52	55.00
YOLOv11s	83.54	57.46	55.44	24.83	58.00
YOLOv11m	86.81	59.28	57.56	24.48	59.00
YOLOv11l	83.91	62.24	61.94	24.81	62.00
**YOLOv11x**	**86.65**	**65.34**	**60.99**	**25.35**	**58.00**

**Table 2:** Comprehensive evaluation of YOLOv5u and YOLOv11 models for rooster-only detection based on body size. Note: Model variants with bold numbers under evaluation metrics represent the best model within each model.

Models	Precision (%)	Recall (%)	mAP@0.50 (%)	mAP@0.50–0.95 (%)	F1-Score (%)
YOLOv5nu	79.07	61.73	62.79	43.19	61.00
YOLOv5su	83.18	69.58	72.84	49.39	70.00
YOLOv5mu	86.49	76.39	80.05	54.75	74.00
YOLOv5lu	86.78	76.06	81.63	56.13	73.00
**YOLOv5xu**	**88.95**	**77.72**	**82.29**	**56.08**	**78.00**
YOLOv11n	81.30	65.89	68.42	47.66	64.00
YOLOv11s	83.50	71.97	75.82	53.23	71.00
**YOLOv11m**	**88.95**	**78.77**	**82.56**	**55.60**	**78.00**
YOLOv11l	87.53	77.20	81.30	56.52	78.00
YOLOv11x	87.43	77.53	82.02	56.70	74.00

## Data Availability

The datasets generated, used, and/or analyzed during the current study will be available from the corresponding author upon reasonable request.
